# Tumour budding in preoperative biopsy specimens is a useful prognostic index for identifying high-risk patients in early-stage (pN0) colon cancer

**DOI:** 10.3906/sag-1903-142

**Published:** 2020-04-09

**Authors:** Mehmet ZENGİN, Aydın ÇİFCİ

**Affiliations:** 1 Department of Pathology, Faculty of Medicine, Kırıkkale University, Kırıkkale Turkey; 2 Department of Internal Medical Sciences, Faculty of Medicine, Kırıkkale University, Kırıkkale Turkey

**Keywords:** Tumour budding, colon cancer, preoperative biopsy, early-stage (pN0)

## Abstract

**Background/aim:**

Tumour budding (BD) is considered a valuable prognostic factor in colon cancer (CC), but its use in daily practice is uncertain. We investigated the prognostic effect of BD using preoperative biopsy specimens in a fairly homogeneous population.

**Materials and methods:**

Eighty-two (pN0) CC patients who underwent surgery after preoperative biopsy between 1997 and 2013 were included in the study. Model A (using the ‘deeply invasive blocks & hot-spot area & invasive margin) and method 1 (using the ‘20× objective & immunohistochemistry staining & quantitive counting’) were used as standard methods.

**Results:**

High BD was significantly associated with poor prognostic factors (lymphatic invasion [P = 0.008], perineural invasion [P = 0.041], advanced pT [P = 0.015], invasive margin [P = 0.008], and margin involvement [P = 0.019]). Moreover, correlations between different BD estimates (r = 0.613–0.696), reproducibility of study (Κappa = 0.68–0.73), and usefulness of cut-off value (area of under ROC = 0.746 [0.663–0.829]) were well. In univariate analysis, 5-year survival was poor in patients with high BD (relaps-free survival [RFS]: 71 %, P < 0.001; overall survival [OS]: 73 %, P = 0.004, local recurrence [LR]: 18 %, P = 0.032). Multivariate analyses confirmed that high BD is an independent worse survival parameter for RFS (Hazard ratio [HR]: 1.53 [1.14–2.80], P = 0.015), OS (HR: 1.44 [1.17–2.75], P = 0.032, and LR (HR: 1.59 [1.05–2.76], P = 0.045).

**Conclusion:**

Our data show that BD provides valuable prognostic information for early-stage (pN0) CC in preoperative biopsy specimens and that adding BD to current risk classification may contribute to better patient selection.

## 1. Introduction 

Colon cancer (CC) is one of the most common cancers in the western world and approximately one-third of patients have early-stage (pN0) disease [1]. Currently, prognosis estimation in CC is performed by the TNM system, which combines histopathological and clinical findings [1,2]. The TNM staging system is widely accepted worldwide, relatively easy, reproducible, and groups patients according to different progress risks [3]. However, even in this system, it is difficult to predict the clinical course individually. This is especially true for early-stage CC patients with a poor 5-year prognosis in approximately 20–30% of patients [4]. Currently, the routine use of adjuvant chemotherapy in this patient population remains unclear. Furthermore, the present risk factors are insufficient to select the ideal patient for adjuvant therapy in this patient population. Therefore, additional prognostic markers are needed for better clinical management [5].

Tumour budding (BD) is defined as the presence of individually and/or in small groups of tumor cells at the invasive front [6]. Many authors think that BD is the first step in epithelial–mesenchymal transition, lymphovascular invasion, lymph node metastasis, and distant organ spread [6,7]. Moreover, several studies have reported that an increase in the number of tumor buds in CC is associated with poor prognosis [8–14]. In addition, the International Tumor Budding Consensus Conference Group recommends that BD be added to high-risk factors in CC [12]. Therefore, this parameter can be a promising index for the detection of high-risk patients in early-stage CCs. However, BD-related studies in the literature show many differences in methodology and few studies have investigated only early–stage and preoperative biopsy [8–14].

In this retrospective cohort, we investigated the predictive value of BD on tumor progression in early-stage (pN0) CC. The distinctive feature of this study was that it represented a fairly homogeneous population and used a standard methodology.

## 2. Materials and methods

**Figure 1:**
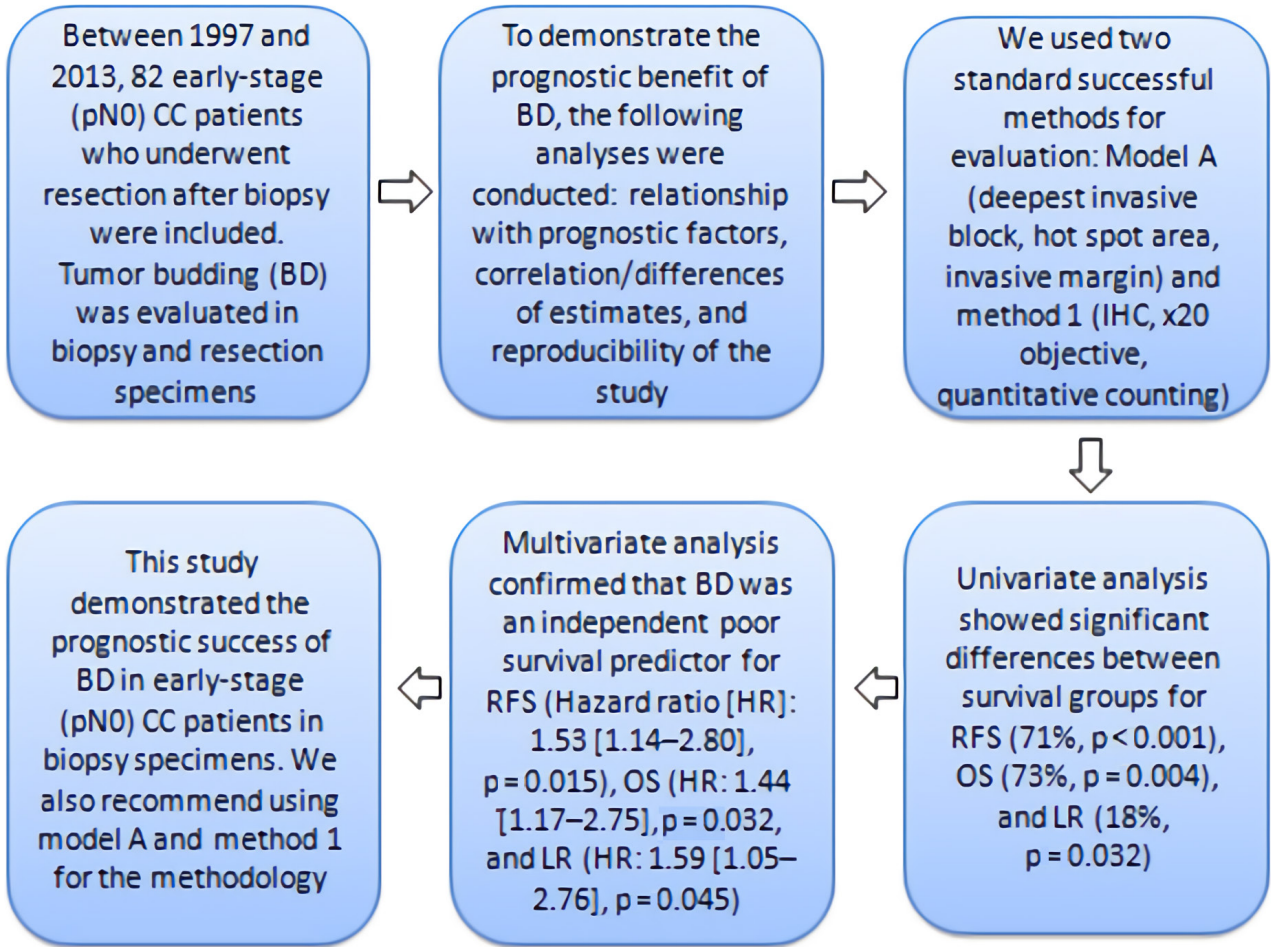
Flowchart of the study CC: colon cancer, IHC: Immunohistochemistry HR: Hazard ratio, OS: Overall survival, RFS: Relapse-free survival

This study was designed according to the recommendations of REMARK [15] and was summarized in Figure 1.

### 2.1. Ethics statement

This study was approved by the Kırıkkale University Health Research Ethics Committee. During this research, attention was paid to comply with the 1964 Helsinki Declaration and the ethical standards of the institutional/national research committee. Informed consent was obtained from each patient and all patients were informed about the content of the study.

### 2.2. Data sources 

This study was performed at a single university hospital in Kırıkkale, Turkey. A total of six hundred and fifty-six patients operated for CC between 1997 and 2013 were included in the study.

### 2.3. Patients 

Retrospective clinical data of the patients were obtained from the archival records of Kırıkkale University. Patients with distant/regional metastasis were not included in this study. Moreover, patients with multiple tumors, secondary tumors, and death/recurrence within 1 month were excluded from the study. Exclusion criteria are summarized as follows: diagnosed with another cancer before/during primary CC (n = 9), without tumor block in archives (n = 8), inadequate tissue for examination (n = 7), stage III and IV disease (n = 530), pN0 disease was not identified in new sections (n​​ = 15), received adjuvant chemoradiotherapy (n = 5). Finally, the study population consisted of eighty-two patients.

### 2.4. Samples

Formol-fixed paraffin-embedded tumor specimens were collected from the archives of Kırıkkale University Department of Pathology. The number of blocks obtained was between 3 and 16 per patient (n = 414, mean = 5.4). Two blocks were selected, one from the preoperative biopsy material and the other from resection materials. For immunohistochemical (IHC) study, attention was paid to the presence of adjacent normal colon tissue and sufficient tumor tissue in the selected blocks. Four 4-µm thick sections (n = 328) were cut from each block, two of them stained with hematoxylin and eosin (H & E), the rest stained with IHC. Pathological evaluation of the primary tumor was performed according to the American Joint Cancer Classification Committee [17]. All sections were evaluated separately by three experienced pathologists and the final value was given according to the average of these observers.

### 2.5. Evaluation of BD 

A bud is defined as a small cluster of adenocarcinomas of up to four cells [16]. The number of tumor buds was visually noted by conventional microscopy (Nikon Eclipse E600, Nikon AG Instruments, Switzerland). 

**Figure 2:**
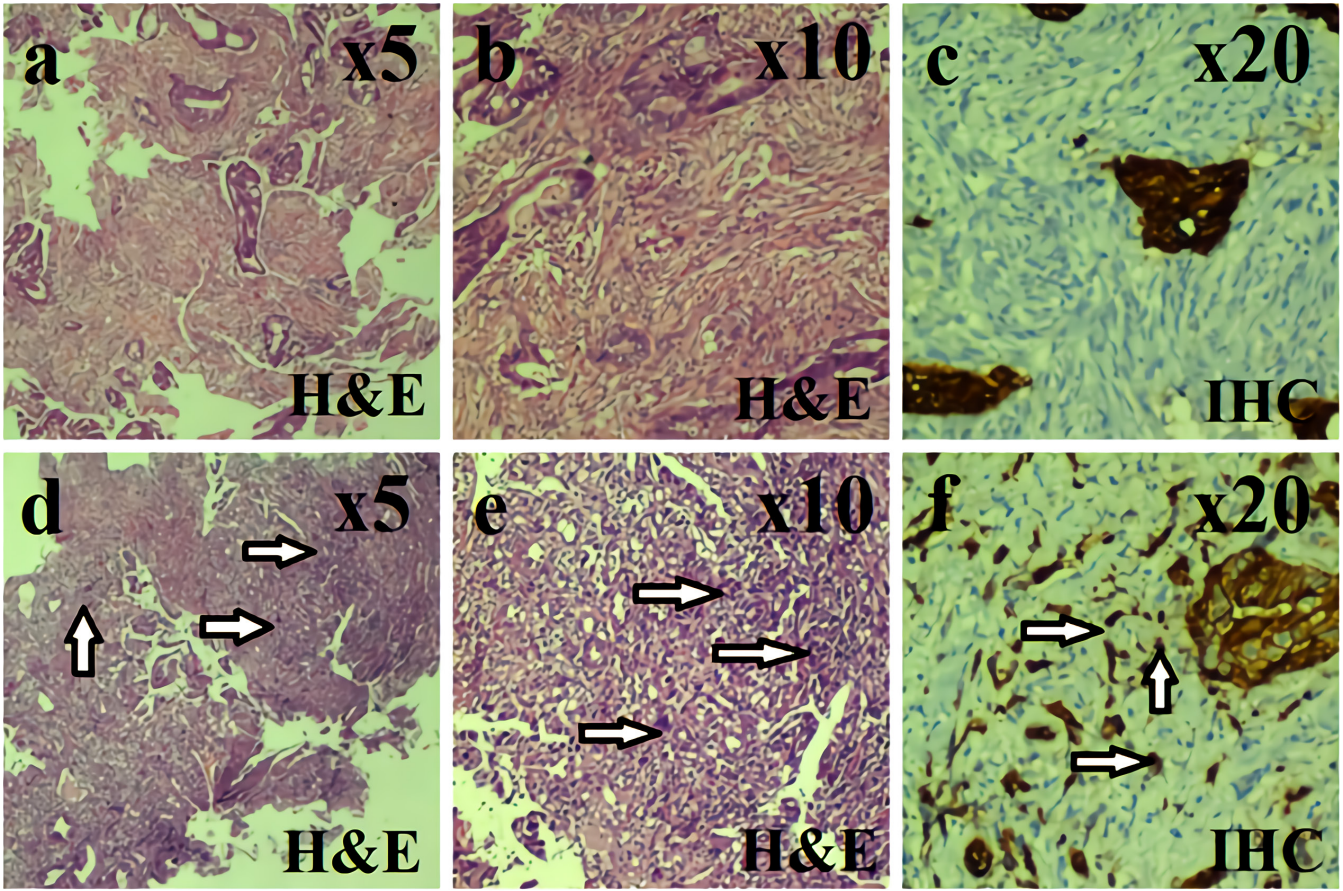
Representative examples for BD counting. We have scanned all the slides using an 10× objective to identify areas with the highest and lowest buds. We chose an area containing mainly tumor buds within the field of view. Tumor buds were present at all borders of the selected image area. We scored BD (arrows) separately with the two methods mentioned above in 10 high power fields. Finally, we divided the cases into two groups as low BD (a-b-c) and high BD (d-e-f). BD: Tumour budding.

Firstly, we scanned all slides using an 10× objective to see the distribution of the tumor buds. Within the field of view, an area containing predominantly tumor buds was selected. It was ensured that the selected buds were present at all borders in this selected image area. Subsequently, BD was separately noted in 10 high-power fields (HPF) according to the methods described above (Figure 2). Finally, all cases were divided into two groups as high-density and low-density according to the optimal cut-off value for survival.

To avoid false IHC staining, adenocarcinoma cells were excluded from the counting unless a clearly defined blue hematoxylin-stained nucleus was present. In sections with less than 10 HPF areas (n = 6), all available HPFs were counted and the final number was given according to the average of these areas.

### 2.6. Optimal evaluation method

One of the most important difficulties in achieving successful results in diagnostic tests is to decide the optimal evaluation method. Many different methods have been used in the literature to evaluate BD [8-14]. This study was based on two successful methods, model A and method 1 [17,18]. Model A recommends using the hot spot area, deepest invasive block, invasive margin. Method 1 recommends the use of immunohistochemistry (IHC) staining, x20 objective, and quantitative counting. Moreover, the optimal cut-off value for a test in clinical studies is usually determined by ROC analysis. The best cut-off value is the value with the lowest false positive rate and with the highest true positive rate. Since the area under a ROC (AUC) curve is usually a measure of the usefulness of a test, a larger area (AUC → 1) means a more useful test [19].

### 2.7. Reproducibility of BD

The reproducibility of the study was evaluated by the following parameters, interobserver agreement and heterogeneity of the tumor. To evaluate these parameters, three independent pathologists scored BD without having the clinical and pathological information. The agreement between the observers was investigated by calculating the weighted and simple Kappa value (ĸ). ĸ value is a ratio of variance indicating interobserver agreement and was classified by Landis et al. [20] as significant, moderate, and excellent for values ​​of 0.41–0.60, 0.61–0.80, and 0.81–1, respectively. Intra- and intertumoral heterogeneity was determined by the Intra-Class Correlation (ICC) test [21]. ICC was considered to be a ratio of the total variance that showed the difference between the tumors examined. If the majority of the variation is due to intertumor variation, e.g., heterogeneity, ICC will be low (ICC → 0), and if the majority of the variation is due to intratumor variation, e.g., biological variation, ICC will be high (ICC → 1).

### 2.8. Patients follow-up 

In this study, survival and recurrence rates were evaluated for outcome measures. Event endpoint time was calculated from the day of primary surgery. The follow-up period was selected as sixteen years (10.5–198.5 months) in all cases. All events after 60 months of follow-up were recorded as 60 months. Relapse-free survival (RFS) was defined as the time from primary surgery to death or local/distant recurrence. Overall survival (OS) was defined as the time between primary surgery day and death or last contact day. The clinical, radiological, and pathological relapse of the disease was called cancer recurrence. This was called local recurrence (LR) if confined to the previous treatment site and was called distant recurrence (DR) if spread to a distant region such as liver and lung.

### 2.9. Immunohistochemical study

Three 4-µm sections (n ​= 246) were cut and placed on a platinum-coated slide of Dako (Denmark, Glostrup, K8020). Pretreatment methods were performed using Dako’s PT link. Using the heat-induced targeting solution of Dako (EnVision Flex), the retrieval epitope was obtained at pH 9, 97 ° C for 20 min. The staining was performed using Dako’s Autostainer link 48. Endogenous peroxidase activity was blocked by Dako’s peroxidase blocking reagent (EnVision Flex). The primary antibody was mouse monoclonal AE1/AE3 (Dako, clone M3515, 1:250) diluted with the antibody diluent of Dako (EnVision Flex). IHC staining of mismatch repair proteins was performed using mouse monoclonal MLH1 (Dako, clone ES05, 1:100) and PMS2 (Dako, clone A16-4, 1:500) antibodies. These antibodies were incubated for 30 min at room temperature and the mouse linker of Dako (EnVision Flex) was used for amplification. The bound antibody was detected by HRP reaction of Dako (EnVision Flex) and visualized by DAB reaction of Dako (EnVision Flex). Meyer hematoxylin (Merck, Germany, Darmstadt) was used for counterstaining and Pertex (Histolab, Sweden, Gothenburg) was used to cover the slides.

### 2.10. Statistical evaluation

Percentage and frequency were used for categorical variables, and range, mean, and standard deviation (SD) were used for continuous variables. Chi-squared test was used for the relationship between clinicopathological features and BD. While analyzing the continuous data, the Wilcoxon signed-level test was used to examine whether there was a difference between these data and Spearman correlation analysis was used to examine whether there was a correlation. As described above, the optimal cut-off value associated with survival was evaluated by the ROC analysis, the heterogeneity of tumors was examined by the ICC test, and the interobserver agreement was investigated by the ĸ test. The difference between univariate survival groups was evaluated by Log-rank test and survival curves were presented by Kaplan-Meier method. Multivariate survival groups were evaluated by Cox-regression model with a 95% confidence interval (CI) and a hazard ratio (HR) of 1.0. All tests were two-sided and P-values less than 0.05 were considered significant. SPSS 21.0 (IBM Institute, North Castle, USA) was used in the analyses.

## 3. Results

### 3.1. Patients

The mean of age and size were 72.48 ± 8.17 years (range: 35–87 years) and 4.67 ± 1.85 cm (range: 2–9 cm), respectively. Thirty-three (40.2 %) of the patients were female and 49 (59.8 %) were male. Thirty-two (39.0 %) of the cases were pT1, 50 (61.0 %) were pT2; 28 (34.1 %) of the cases were low/moderately differentiated, and 54 (65.9 %) were poorly differentiated. 

### 3.2. Scoring of BD 

In BD screening, the distribution of buds was not homogeneous on the slides. One independent section with a good bud homogeneity level was selected from preoperative and postoperative biopsy samples. The mean of BD numbers was 7.37 ± 4.84 for the biopsy and was 7.98 ± 5.24 for the resection, respectively. Representative images for BD counting were shown in Figure 2.

### 3.3. Optimal evaluation method

**Figure 3:**
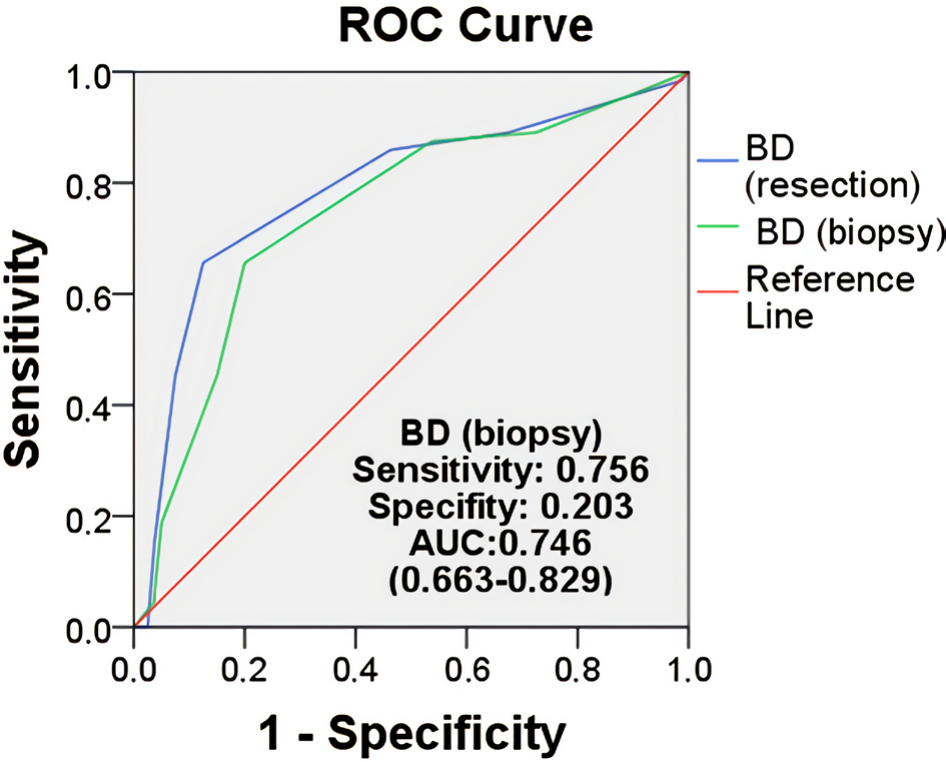
Optimal cut-off value for BD. AUC analyzed by manual methods. BD: Tumour budding, ROC: Receiver Operating Characteristic, AUC: Areas under the ROC curves.

BD was scored separately using model A and method 1 as described above. When the results were examined, there was a good relationship between BD (biopsy) and poor prognostic parameters (lymphatic invasion [P = 0.008], perineural invasion [P = 0.041], advanced pT [P = 0.012], invasive margin [P = 0.008] and margin involvement [P = 0.019]) (Table 1). Moreover, when continuous data were analyzed, the correlation between BD (biopsy) estimates was quite high (R = 0.696, P < 0.001) and the difference was quite low (R = 0.321, P < 0.001) (Table 2). In addition, the cut-off value for BD (biopsy) was useful (ROC: 10.37; AUC = 0.746 [0.663-0.829]) (Figure 3). For convenience, this value was considered 10 and all samples were divided into two groups using this value.

**Table 1:** Relationship between BD and prognostic factors.

		Biopsy			Resection		
		Low BD	High BD	P-value	Low BD	High BD	P-value
Age				0.432			0.135
	<72	15 (53)	24 (44)		15 (60)	24 (42)	
	≥72	13 (47)	30 (56)		10 (40)	33 (58)	
Size				0.119			0.067
	<4 cm	17 (60)	23 (42)		16 (64)	24 (42)	
	≥4 cm	11 (40)	31 (58)		9 (36)	33 (58)	
Gender				0.281			0.313
	Female	9 (32)	24 (44)		8 (32)	25 (43)	
	Male	19 (68)	30 (56)		17 (68)	32 (57)	
Lymphatic invasion				0.008*			0.014*
	No	19 (67)	20 (37)		17 (68)	22 (38)	
	Yes	9 (33)	34 (63)		8 (32)	35 (62)	
Perineural invasion				0.041*			0.022*
	No	17 (60)	20 (37)		16 (64)	21 (36)	
	Yes	11 (40)	34 (63)		9 (36)	36 (64)	
LIR				0.759			0.925
	No	15 (53)	27 (50)		13 (52)	29 (51)	
	Yes	13 (47)	27 (50)		12 (48)	28 (49)	
pT-stage				0.015*			0.009*
	pT1	16 (57)	16 (29)		15 (60)	17 (29)	
	pT2	12 (43)	38 (71)		10 (40)	40 (71)	
Invasivemargin				0.008*			0.002*
	No	20 (71)	22 (40)		19 (76)	23 (40)	
	Yes	8 (29)	32 (60)		6 (24)	34 (60)	
Margin involvement				0.019*			0.008*
	No	19 (67)	22 (40)		18 (72)	23 (40)	
	Yes	9 (33)	32 (60)		7 (28)	34 (60)	
MSI Status				0.275			0.386
	MMR-P	16 (57)	24 (44)		14 (56)	26 (45)	
	MMR-D	12 (43)	30 (56)		11 (44)	31 (55)	
Grade				0.782			0.459
	Low-grade	9 (50)	19 (44)		10 (40)	18 (31)	
	Moderate / High-grade	19 (50)	35 (56)		15 (60)	39 (69)	
Tumour necrosis				0.351			0.258
	No	16 (57)	25 (46)		13 (52)	22 (38)	
	Yes	12 (43)	29 (54)		12 (48)	35 (62)	

*. The significance level for the P-value is 0.05. Significant results are shown in italics. BD: Tumour budding, LIR: Local inflammatory response, MSI: Microsatellite instability, MMR-P: Mismatch repair proteins proficiency, MMR-D: Mismatch repair proteins deficiency, pT: Pathologic tumour stage

**Table 2:** Analysis of continuous variables for BD.

	N	BD (Biopsy)	BD (Resection)
BD (A & B)	82	0.696 (S), P = 0.321 (W)	0.729 (S), P = 0.312 (W)
BD (A & C)	82	0.642 (S), P = 0.435 (W)	0.686 (S), P = 0.438 (W)
BD (B & C)	82	0.613 (S), P = 0.473 (W)	0.617 (S), P = 0.470 (W)

BD: Tumour budding, S: Spearsman correlation analysis, W: Wilcoxon Signed Rank test, A: First observer, B: Second observer, C: Third observer, N: Number

### 3.4. Reproducibility of BD

The analysis was performed for both categorical and continuous variables and similar results were found. Therefore, only the best results were given here as an example. The reproducibility of the study was evaluated as follows: 

#### 3.4.1. Agreement of observers

In general, the interobserver agreement ranged from moderate to significant and was clinically useful (ĸ = 0.68–0.73). We also found that the interobserver agreement for BD (biopsy) was slightly lower than BD (resection) (Table 3). This was a finding we expected. Because a smaller area was examined in the biopsy material compared to the resection material.

#### 3.4.2. Heterogeneity of tumor

In general, the majority of the variation was due to biological differences between tumors. For example, an ICC count of 0.677 means that 67.7% of the total variance is due to intertumor heterogeneity. Moreover, ICC values ​​of BD (biopsy) were slightly lower than BD (resection). This can be explained as follows. As more areas of the tumor were examined, heterogeneity was higher in resection materials (Table 3).

**Table 3:** Reproducibility of study.

	N	ICC- Categorical (95 % CI)	ĸ values
BD (A & B) (Resection)	82	0.677 (0.584-0.803)	0.73
BD (A & C) (Biopsy)	85	0.654 (0.532-0.785)	0.71
BD (B & C) (Resection)	82	0.638 (0.513-0.771)	0.68

BD: Tumour budding, ĸ: Kappa values, ICC: Intra-class correlation coefficient, CI: Confidence interval, A: First observer, B: Second observer, C: Third observer, N: Number

### 3.5. Follow-up events

Twenty-seven patients died (high BD, n = 25; low BD, n = 2) during the 16-year follow-up and twenty-nine patients recurred (high BD, n = 26; low BD, n = 3). Moreover, twelve patients had LR (high BD, n = 10; low BD, n = 2) and ten patients had DR (high BD, n = 8; low BD, n = 2). Five-year RFS and OS ratios were 71–73% in high BD (biopsy) patients and 95–95% in low BD (biopsy) patients, respectively. Moreover, five-year LR and DR ratios were 18–16% in high BD (biopsy) and 6–7% in low BD (biopsy), respectively (Table 4). 

**Table 4:** Univariate survival analysis of BD.

		OS		RFS		LR		DR	
		5-year (%)	P-value	5-year (%)	P-value	5-year (%)	P-value	5-year (%)	P-Value
Age			0.736		0.644		0.686		0.744
	< 72	88		89		14		14	
	≥ 72	82		81		16		16	
Size			0.415		0.384		0.461		0.512
	< 4 cm	90		91		12		13	
	≥ 4 cm	80		80		16		15	
Gender			0.878		0.792		0.835		0.962
	Female	87		88		15		15	
	Male	83		82		14		15	
Lymphaticİnvasion			0.257		0.182		0.212		0.374
	No	91		92		10		11	
	Yes	78		78		15		15	
Perineuralİnvasion			0.229		0.147		0.353		0.467
	No	92		92		12		12	
	Yes	77		77		15		16	
LIR			0.819		0.718		0.844		0.954
	No	87		87		15		16	
	Yes	84		83		16		16	
pT-stage			0.068		0.033*		0.199		0.286
	pT1	93		94		8		9	
	pT2	75		72		16		16	
Invasivemargin			0.274		0.156		0.465		0.779
	No	91		80		12		14	
	Yes	78		84		15		15	
Margininvolvement			0.042*		0.014*		0.130		0.176
	No	94		95		11		11	
	Yes	74		71		16		16	
MSI Status			0.866		0.722		0.719		0.945
	MMR -P	86		86		16		15	
	MMR -D	83		85		14		15	
Grade			0.945		0.894		0.831		0.714
	Low grade	85		85		15		14	
	Moderate /High grade	84		84		16		16	
Tumournecrosis			0.524		0.461		0.458		0.572
	No	86		88		13		13	
	Yes	85		84		15		16	
BD(Biopsy)			0.004*		0.001*		0.032*		0.065
	Low	95		96		6		7	
	High	73		71		18		16	
BD(Resection)			0.001*		<0.001*		0.018*		0.042*
	Low	96		96		5		6	
	High	72		70		18		17	

*. The significance level for the P-value is 0.05. Significant results were in italics. Abbreviations: BD: Tumour budding, pT: Pathologic tumour stage, LIR: Local inflammatory response, MSI: Microsatellite instability, MMR-P: Mismatch repair proteins proficiency, MMR-D: Mismatch repair proteins deficiency, OS: Overall survival, RFS: Relapse-free survival, LR: Local recurrence, DR: Distant recurrence

### 3.6. Univariable survival analyses

**Figure 4:**
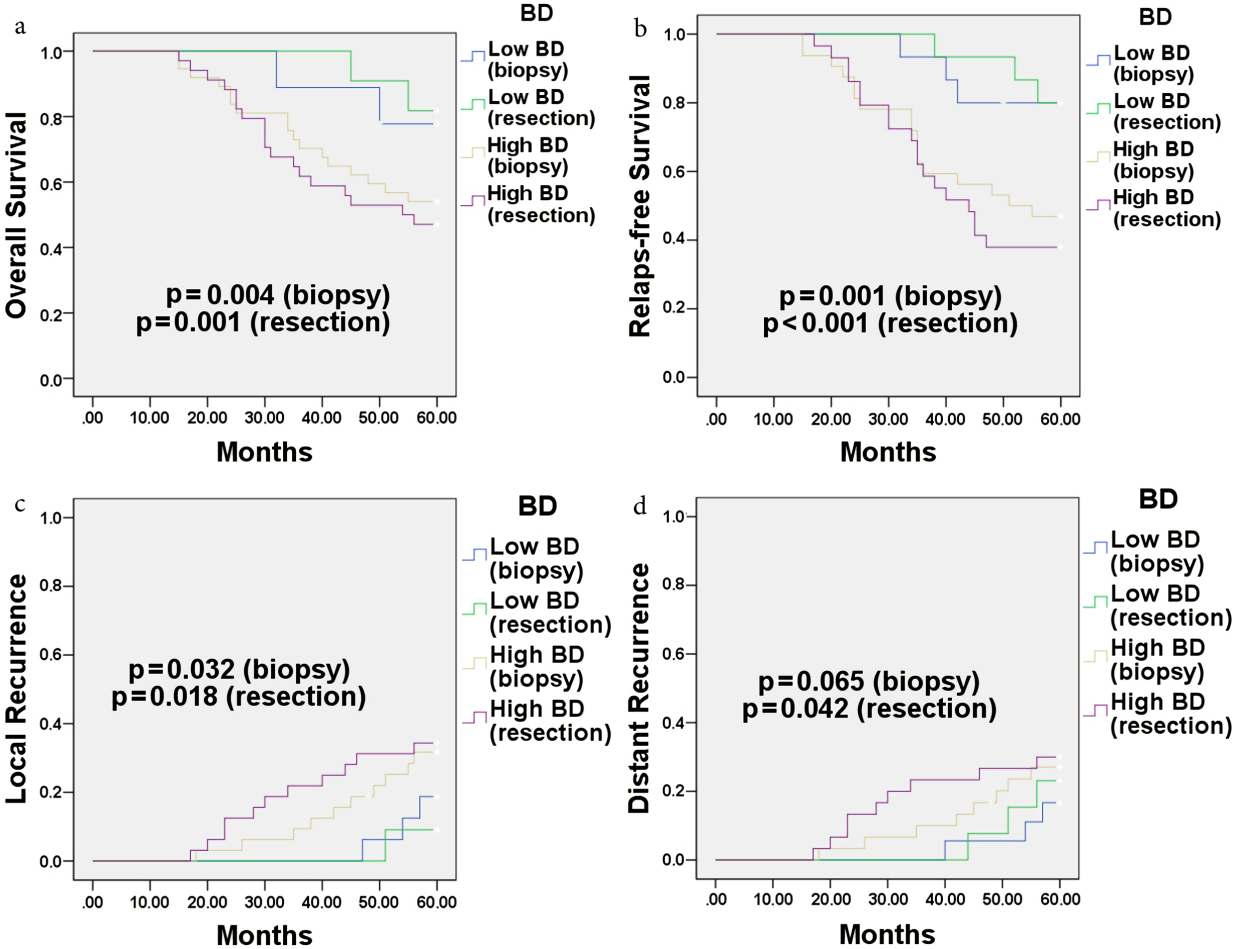
Survival and recurrence curves for BD. Kaplan–Meier survival curves were used for overall survival (a), relapse-free survival (b), local recurrence (c), and distant recurrence (d). The significance level for the P-value is 0.05

In univariate analysis, significant differences were observed between BD (biopsy) and survival groups for RFS (P = 0.001), OS (P = 0.004), and LR (P = 0.032). Moreover, pT-stage and margin involvement were significantly associated with poor RFS and margin involvement was significantly associated with poor OS (Table 4, Figure 4). 

### 3.7. Multivariable survival analyses

In multivariate analysis, high BD (biopsy) was an independent worse prognostic parameter for RFS (HR = 1.53 [1.14–2.80], P = 0.015), OS (HR = 1.44 [1.17–2.75], P = 0.032 ), and LR (HR = 1.59 [1.05–2.76], P = 0.045). Margin involvement was another parameter that was significantly associated with poor RFS (Table 5). 

**Table 5:** Multivariate survival analysis of the four parameters.

		Overall survival(n = 82) (%)		Relaps-free survival(n = 82) (%)		Local recurrence(n = 82) (%)		Distant recurrence(n = 82) (%)	
		HR(95 % CI)	P value	HR (95 % CI)	P value	HR(95 %CI)	P value	HR(95 % CI)	P value
pT-stage	pT1	1	-	1	-	1	-	1	-
	pT2	2.57(0.65-10.7)	0.356	1.86(0.55-11.1)	0.284	3.81(0.50-7.83)	0.519	NC	0.897
Margin involvement	No	1	-	1	-	1	-	1	-
	Yes	2.35(0.57-9.33)	0.212	1.63(1.19-2.91)	0.041*	7.60(0.44-13.5)	0.453	9.30(0.39-17.4)	0.594
BD	<10	1	-	1		1	-	1	-
(Biopsy)	≥10	1.44(1.17-2.75)	0.032*	1.53(1.14-2.80)	0.015*	1.59(1.05-2.76)	0.045*	1.67 (0.91-3.12)	0.098
BD	<10		-	1	-	1	-	1	-
(Resection)	≥10	1.42(1.23-2.89)	0.013*	1.49(1.17-2.64)	0.003*	1.57(1.07-2.54)	0.033*	1.65 (0.93-3.24)
0.056

*. The significance level for the P-value is 0.05. Significant results in italics. BD: Tumour budding, pT: Pathologic tumour stage, CI: Confidence interval, HR: Hazard ratio, OS: Overall survival, RFS: Relapse-free survival, LR: Local recurrence, DR: Distant recurrence, NC: Not calculable

## 4. Discussion

In this study, we investigated the prognostic effect of BD in early-stage (pN0) CC patients who underwent surgery after the preoperative biopsy. Our results show that the evaluation of this factor in preoperative biopsies is useful in predicting prognosis. We also found that the use of model A and method 1 is beneficial in the evaluation of BD.

For preoperative biopsy specimens, BD has been shown to be a poor prognostic factor. For example, Morodomi et al. [22] showed that the budding number was associated with lymph node metastasis in rectal cancer patients. Giger et al. [23] examined preoperative biopsies and corresponding resection specimens in colorectal cancer patients and confirmed these findings. Rogers et al. [24] demonstrated the predictive power of BD for nodal metastasis in rectal cancer patients treated with neoadjuvant therapy. However, these studies are quite different in terms of both assessment methods and study populations. In this study, we found that BD is an independent prognostic factor for poor RFS, OS, and LR. Moreover, the population consisted of only early-stage patients (pN0) and only CC patients. In addition, to increase the homogeneity of the population, patients treated with adjuvant chemotherapy and known to have secondary malignancy were excluded. In other words, in contrast to other studies, we selected our patient population to be highly homogeneous.

For resection specimens, many early-stage CC studies in the literature have shown that high BD is associated with worse prognosis [25–30]. Moreover, a few studies have found no prognostic significance [31]. The main reason why this prognostic marker cannot be fully integrated into pathology reports is the lack of a standardized evaluation system [25–30]. A different feature of this study is that it provides a standard approach to pathological evaluation. That is, two standard evaluation methods were used in this study. Briefly, histopathological evaluations can be divided intrabiopsy evaluation (section, area and focus) and extrabiopsy evaluation (staining, magnification, and counting). Model A [17] was used for intrabiopsy evaluation and method 1 [18] was used for extrabiopsy evaluation. And both of these methods yielded successful results. Therefore, unlike other studies, our study was quite standard in terms of methodology.

There are different findings regarding the ratio and mean value of BD in publications. In general, high BD rates of 19% to 45% [8,32] and mean bud values of 7.11 to 8.05 [25,32] have been reported. For example, Koelzer et al. [25] reported a high BD rate of 30% and an average of 7.11 buds. We found a high BD rate of 65% and an average of 7.37 buds. These differences can be explained by the heterogeneity of tumors and the variety of evaluation methods. In the following paragraph, we will discuss the heterogeneity of BD. As for the differences in evaluation methods, we used two successful standard methods described above in this study. Moreover, we calculated BD in 10 HPFs, and this method can change the average number. In addition, we have only counted BD cells with a clearly identifiable blue nucleus, so the results may have changed due to this counting rule. As a result, we believe that the differences arise from the variability of the methods, and we recommend the above-mentioned standard counting technique for future studies.

In the literature, the issue of heterogeneity of CC is considered a serious problem [33,34]. For example, Mesker et al. [33] reported that the deeply infiltrated tumor sections in the bowel wall had the lowest tumor cells and recommended the use of the highest pT-stage histological section in the evaluation of the primary tumor. In this study, we used the deeply infiltrated tumor section and we found that the heterogeneity of BD was significantly higher among different tumors. We believe that this problem can be overcome by the two standard methods mentioned above. Moreover, it is understood that different technical approaches can provide a higher degree of precision and accuracy. In future studies, the heterogeneity of CC needs to be further investigated methodologically.

The current consensus in the literature suggests that BD should be evaluated using H & E [11,12]. However, there are also studies reporting that evaluation of BD with IHC increases detection rates and interobserver agreement [25,35]. However, it is not clear whether the evaluation by IHC is prognostically different from the evaluation by H & E. In our study, although the evaluation was mainly made with IHC stained sections, we also evaluated the H & E stained sections at some stages of our study. One of the challenges of using IHC was as that some cell types other than malignant adenocarcinoma cells also showed reactivity with IHC, e.g., cells of vascular neoangiogenesis. One of the difficulties in using H & E was that many different structures had a budding-like appearance, e.g., disintegration of tumor glands secondary to intense inflammation. As a result, more comprehensive studies are needed for standardization of staining methods.

There are many important aspects of our research. A good parameter recently discussed in numerous large studies was presented. Our population was quite homogeneous because it was based on a well-characterized cohort of early-stage (pN0) CC patients without adjuvant therapy. Two well-standardized pathological methods were used in this study. And all stages of this study were designed according to the REMARK guidelines.

Our study had some limitations. First, it was impossible to overcome the sampling difference since the tissue under investigation was sampled for diagnosis previously. We have evaluated many different areas of a tumor, but we know that this was only a small part of an entire tumor. Recurrence and death data were obtained from archive records and individual patient records were not evaluated. Moreover, since patients were treated according to protocols before 2013, there may be differences with current treatment protocols.

Our results confirm the predictive value of BD in CC patients. At least hypothetically, BD can predict the need for chemo-radiotherapy in early-stage (pN0) patients in preoperative biopsy specimens. We also recommend using model A and method 1 for more successful results in future studies.

## Acknowledgments

We are grateful to the members of the Department of Pathology and Internal Medicine for their sincere support and participation.
